# Radiation dose fractionation and its potential hormetic effects on male *Glossina palpalis gambiensis* (Diptera: Glossinidae): a comparative study of reproductive and flight quality parameters[Fn FN1]

**DOI:** 10.1051/parasite/2024001

**Published:** 2024-02-08

**Authors:** Bénéwendé Aristide Kaboré, Syeda Dua Taqi, Athumani Mkinga, Anibal E Morales Zambrana, Robert L Mach, Marc JB Vreysen, Chantel J de Beer

**Affiliations:** 1 Joint FAO/IAEA Centre of Nuclear Techniques in Food and Agriculture, International Atomic Energy Agency, Vienna International Centre 1400 Vienna Austria; 2 Institute of Chemical, Environmental and Bioscience Engineering, Vienna University of Technology, Gumpendorfer Straße 1a 1060 Vienna Austria; 3 Insectarium de Bobo-Dioulasso-Campagne d’Eradication de la mouche Tsétsé et de la Trypanosomose Bobo-Dioulasso BP 1087 Burkina Faso; 4 Vector and Vector-Borne Diseases Institute, Tanzania Veterinary Laboratory Agency 1026 Tanga Tanzania

**Keywords:** Tsetse pupae, Gamma-ray irradiation, Sterility, Flight propensity, Sterile insect technique

## Abstract

One of the most critical factors for implementing the sterile insect technique for the management of tsetse is the production of large quantities of highly competitive sterile males in the field. Several factors may influence the biological quality of sterile males, but optimizing the irradiation protocols to limit unwanted somatic cell damage could improve male performance. This study evaluated the effect of fractionation of gamma radiation doses on the fertility and flight quality of male *Glossina palpalis gambiensis*. Induced sterility was assessed by mating irradiated males with virgin fertile females. Flight quality was assessed using a standard protocol. The male flies were irradiated as pupae on day 23–27 post larviposition with 110 Gy, either in a single dose or in fractionations of 10 + 100 Gy and 50 + 60 Gy separated by 1-, 2- and 3-day intervals or 55 + 55 Gy separated by 4-, 8-, and 24-hour intervals. All treatments induced more than 90% sterility in females mated with irradiated males, as compared with untreated males. No significant differences were found in emergence rate or flight propensity between fractionated and single radiation doses, nor between the types of fractionations. Overall, the 50(D0) + 60(D1) Gy dose showed slightly higher induced sterility, flight propensity, and survival of males under feeding regime. Dose fractionation resulted in only small improvements with respect to flight propensity and survival, and this should be traded off with the required increase in labor that dose fractionation entails, especially in larger control programs.

## Abstract (fr)

L’un des facteurs les plus critiques pour la mise en œuvre de la technique de l’insecte stérile pour la gestion des glossines est la production de grandes quantités de mâles stériles hautement compétitifs sur le terrain. Plusieurs facteurs peuvent influencer la qualité biologique des mâles stériles, mais l’optimisation des protocoles d’irradiation pour limiter les dommages indésirables aux cellules somatiques pourrait améliorer les performances des mâles. Cette étude a évalué l’effet du fractionnement de la dose d’irradiation gamma sur la fertilité et la qualité de vol des mâles de *Glossina palpalis gambiensis*. La stérilité induite a été évaluée en accouplant des mâles irradiés avec des femelles vierges et fertiles. La qualité du vol a été évaluée à l’aide d’un protocole standard. Les mouches mâles ont été irradiées sous forme de pupes agées de 23 à 27 jours après la larviposition avec 110 Gy, soit en dose unique, soit en fractions de 10 + 100 Gy et 50 + 60 Gy séparées par 1, 2 et 3 jours ou 55 + 55 Gy séparés par des intervalles de 4, 8 et 24 heures. Tous les traitements ont induit plus de 90 % de stérilité chez les femelles accouplées avec des mâles irradiés par rapport aux mâles non traités. Aucune différence significative n’a été trouvée dans le taux d’émergence ou la propension au vol entre les doses d’irradiation fractionnées et uniques ni entre les types de fractionnements. Dans l’ensemble, la dose de 50 (J0) + 60 (J1) Gy a montré une stérilité induite, une propension à voler et une survie légèrement plus élevées chez les mâles sous régime alimentaire. Le fractionnement de dose n’a entraîné que de légères améliorations en ce qui concerne la propension à voler et la survie, et cela devrait être compensé par l’augmentation nécessaire du travail qu’implique le fractionnement de dose, en particulier dans les programmes de contrôle de grande envergure.

## Introduction

Trypanosomes are parasitic protozoa that cause the debilitating diseases African Animal Trypanosomosis (AAT) in livestock, which is also known as Nagana, as well as Human African Trypanosomosis (HAT), also known as sleeping sickness in humans. AAT and HAT are mainly transmitted by tsetse flies, the sole cyclical vectors in sub-Saharan Africa [39, 63] where they are distributed over approximately 10 million km^2^ [43]. The disease causes significant losses in animal production and makes fertile land inaccessible for cultivation. As a result, it is responsible for huge economic losses, making it a major limiting factor for sustainable agricultural development in affected areas [17, 35]. Thus, several methods have been developed to control the disease and its vectors.

One of the control methods is the sterile insect technique (SIT), a method of pest control that involves the release of large numbers of sterilized insects, usually males, to compete with wild males for mating opportunities with wild females. The released sterile males mate with wild females and subsequently no offspring is produced, resulting in a reduction of the pest population over time [24]. The SIT is implemented as part of an area-wide integrated pest management (AW-IPM) program where it is integrated with other control measures to manage an entire pest population in a circumscribed area. Control tactics that can be integrated with the SIT, and that are currently acceptable from an environmental point of view, are the sequential aerosol technique (SAT), the use of traps and insecticide impregnated targets, and the live bait technique. The SIT has been successfully used against various insect pests species [32, 36, 53, 64], including tsetse flies in central Nigeria [51], in Sidéradougou (Burkina Faso) [47], on the Island of Unguja, Zanzibar [57], and in the Niayes of Senegal [58].

In all past tsetse programs that had an SIT component, producing the required quantities of sterile males was one of the main challenges. The SIT can only be successful when the colony-reared and released sterile males are as competitive as their wild counterparts to mate with wild females [61]. To produce high-quality sterile males for use in SIT programs, factors and processes such as the rearing, sterilization, transport, handling and release must be considered and properly managed as they are the critical factors that can affect the quality of the sterile males [66, 68].

To address these challenges, programs that have an SIT component continuously aim at improving the protocols for insect rearing and handling, shipment, and irradiation processes. Improvement of irradiation protocols can include insects live stage radiation sensitivity refinements, radiation under various atmosphere and environmental conditions, or dose fractionation. Several studies have investigated the effects of irradiation under different conditions on the quality of sterile males produced for SIT programs. Some of these studies have shown that irradiation under nitrogen (hypoxia or anoxia) or other modified atmospheres can improve the quality of the sterile males [10]. Other studies based on dose fractionation showed the same trend with improved male performance, *i.e.*, induced sterility, longevity, or mating competitiveness in insect pests of crops, livestock, and vectors of human diseases [52]. Fractionation of the radiation doses applied to the Indian meal moth, *Plodia interpunctella* showed a significant improvement of male longevity, mating competitiveness, and sterility [14], and similarly, a significant improvement was obtained in male *Aedes aegypti’s* survival and mating competitiveness [67]. Dose fractionation applied to *Glossina morsitans* increased their residual fertility as compared with a similar but continuous single dose [21]. Early stage pupae of *Glossina tachinoides* that were irradiated with fractionated and single doses under nitrogen atmosphere resulted in a similar induced sterility but survival was better [59]. Therefore, administering split doses lower than the optimal dose, separated by an ideal time interval, could induce a hormetic effect, resulting in the improvement of such parameters [56].

As only a limited number of studies on the effect of dose fractionation on tsetse quality have been carried out, the effect of an optimal dose of 110 Gy fractionated into 10 + 100 Gy and 50 + 60 Gy, separated by 1-, 2-, and 3-day intervals or a fractionated doses of 55 + 55 Gy separated by 4-, 8-, and 24-hour intervals, as compared with a single dose, was investigated on male *Glossina palpalis gambiensis*.

## Materials and methods

### Tsetse strain, rearing and samples selection

A *G. p. gambiensis* strain has been maintained at the Insect Pest Control Laboratory (IPCL) of the Joint FAO/IAEA Centre of Nuclear Techniques in Food and Agriculture, Seiberdorf, Austria since 2009. The colony was established from pupae provided by the Centre International de Recherche-Développement sur l’Élevage en zone Subhumide (CIRDES) in Bobo Dioulasso, Burkina Faso. The original colony was established at Maisons-Alfort (France) in 1972 from wild pupae collected in Guinguette (Burkina Faso, Latitude: 11.56165885, Longitude: – 4.16220681) and transferred to CIRDES in 1975 [41]. In 1981, additional wild pupae collected in Mare aux Hippopotames (Burkina Faso, Latitude: 11.2043219, Longitude: – 4.4382593) were introduced into the colony.

The colony, experimental pupae, and flies were maintained under standard laboratory conditions in a climate-controlled room at a constant temperature of 24 ± 1.0 °C, a relative humidity (RH) of 75–80%, and under subdued/indirect illumination in a 12 h light/12 h dark photoperiod [26, 30]. The colony flies were offered a blood meal of defibrinated bovine blood three times per week, using a TPU 4 *in vitro* feeding system [30]. Males and females of the flies in experiment 1 were fed manually at the same frequency.

Pupae were collected twice daily (9:00 and 15:00) and incubated at 24.0 ± 1.0 °C and 75.0 ± 5.0 RH. The pupae were then sex sorted with the newly developed Near Infrared Pupae Sex Sorter (NIRPSS) 23–24 days post larviposition based on the melanization ratio (unmelanized pupae/total pupae sorted) [2]. Male pupae were selected from the pupae classified as non-melanized when the unmelanized ratio was below 35%, whereas the melanized pupae were returned to the colony.

### Irradiation procedures and dosimetry

A Foss 812 gamma irradiator (Foss Therapy Services Inc., Pacoima, CA, USA) was used to assess the effects of dose fractionation on male *G. p. gambiensis* pupae. This Co-60 Self-Contained Irradiator comprises three sources and three turntables, yielding a combined power of 22,500 Curies per operation. The irradiation setup was executed by activating all three sources and utilizing turntable 3. This set up involved a dose rate decline from 69.49 Gy/min at the onset of the experiment to 62.15 Gy/min at the end. The canister set-up was based on a Plexiglas tube attached to double 9 cm × 9 cm × 1.5 cm Petri dishes so that the sample in a small plastic vial was inserted into the tube to be in the central position of the irradiation chamber. The GAFchromic dosimetry system, with HD-V2 for the high and MD-V3 for the low doses, was used according to the IAEA standard operating procedures [27]. During each irradiation, three MD or HD films placed in 2 cm × 2 cm paper envelopes were included in the sample vial. An optical density reader (wavelengths of 458 nm and 590 nm) was used to read the irradiated films 24 hours after irradiation.

**Figure 1:**
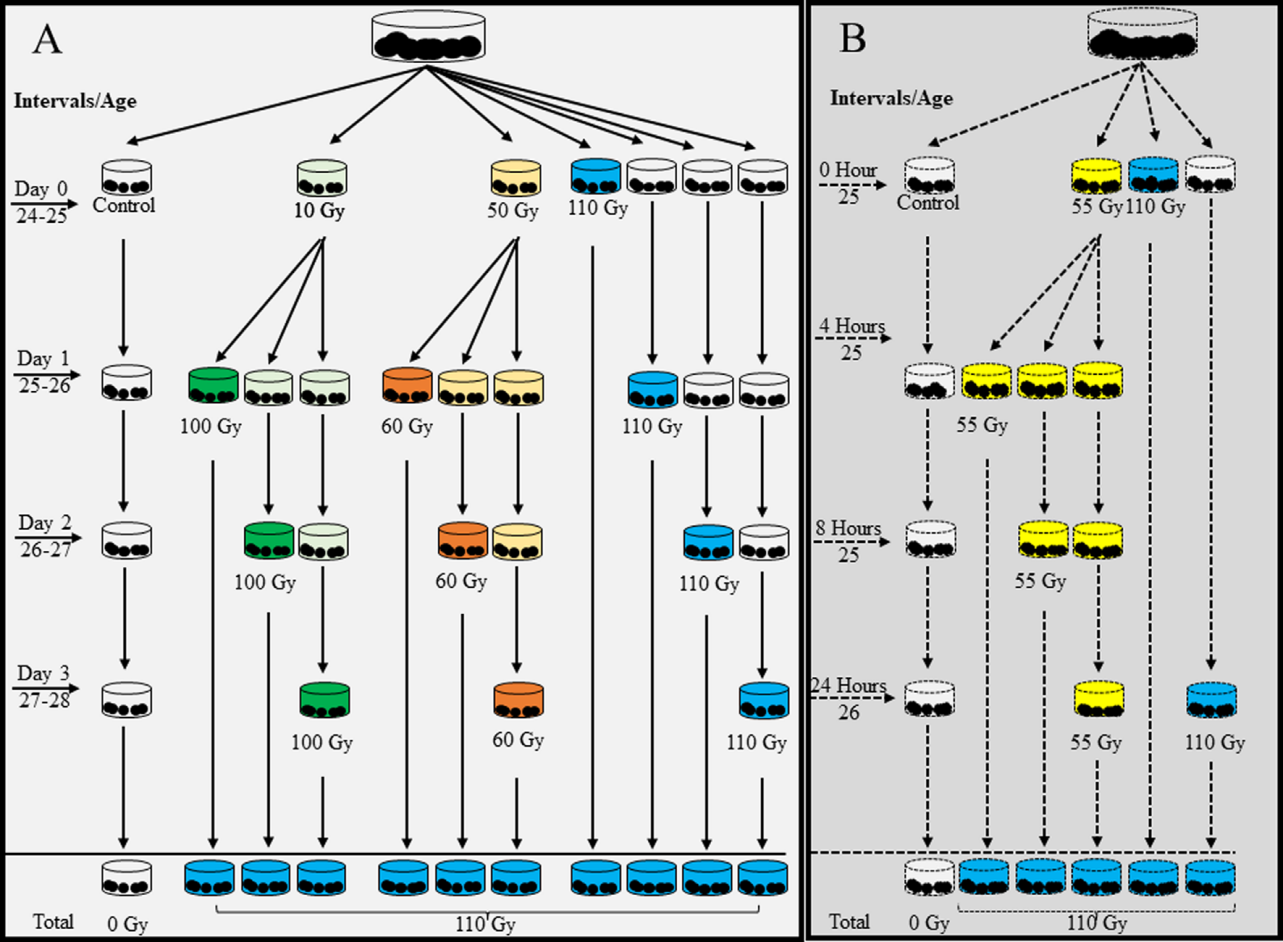
Flowchart of fractionation procedures for the irradiation *of male Glossina palpalis gambiensis* pupae. Pupae were irradiated with dose of 110 Gy, administered as a single dose or fractionated into 10 + 100 Gy and 50 + 60 Gy separated by 1-, 2- and 3-day intervals (A) or using equal fractionated doses of 55 + 55 Gy separated by 4-, 8- and 24-hour intervals (B). The first fraction was administered on day zero/hour zero, and the second fraction on days 1, 2 and 3, or 4, 8 and 24 hours later. The control pupae that were not irradiated were handled in the same conditions.

Previous dose response experiments with *G. p. gambiensis* pupae using both gamma and X-rays indicated that 110 Gy was sufficient to induced more than 97% sterility in females [54, 65]. For the treatment groups, *G. p. gambiensis* pupae were therefore exposed to a total dose of 110 Gy. First, two types of fractionations, *i.e.*, 10 + 100 Gy and 50 + 60 Gy separated by 1, 2 and 3 days were evaluated in comparison with single doses following the same time interval (Fig. 1A). Pupae were exposed to irradiation on days 23–27 post larviposition. An additional experiment was conducted after reviewing the results from the small and nearly half-fractionations. Pupae of 25 days old were exposed to 55 + 55 Gy administered with 4-, 8-, and 24-hour intervals, in comparison with exposure to a single dose of 110 Gy administered on day 0 and 24 h later (Fig. 1B). All replicates had a control group of pupae which was not irradiated and subjected to the same environmental conditions.

### Assessing adult emergence rate, survival under feeding regime, and induced sterility

After irradiation, control and irradiated pupae were transferred to 35 mm diameter Petri dishes and maintained in emergence cages in an incubation room. At emergence, only males were maintained and fed under the conditions described above.

For each treatment, 6–7-day-old males were mated with 3–4-day-old virgin females at 1:1 ratio or slightly less, male (*n* = 1,265) to female (*n* = 1,430), in standard colony cages (20 cm Ø). After 4 days, males and females were separated under low temperature (4 °C) conditions. The females were kept in standard colony cages and placed on individual dishes, whereas the males were transferred into small cages (110 mm Ø). Daily observations were made of male (90 days) and female (60 days) mortality as well as pupae production and egg/larvae abortions. Females were dissected when they were 60 days old to determine their reproductive status. The content of the spermathecae was scored as 0 (empty), 0.25 (quarter), 0.50 (half), 0.75 (three-quarters) or 1 (full), and the content of the uterus was examined to determine the reproductive stage that could be a post-larviposition/blockage or the discovery of aborted eggs and immature larval stages (I, II, III). This experiment was replicated five times.

### Evaluating flight quality using fractionated doses: adult emergence rate, flight propensity, and survival under stress regime

All treatment groups described in Figures 1A and 1B were used for the flight quality control test, as described by Seck *et al.* [50].

Pupae were placed in small Petri dishes (35 mm Ø) in the center of 90 mm Ø petri dishes surrounded by a black cylinder (10 cm high and 9.4 cm Ø). To prevent the emerging flies from crawling out of the cylinder, the inner walls were coated with unscented talc. The flight tube with pupae was then kept in a BugDorm cage of 30 cm × 30 cm × 30 cm (MegaView Science Co., Ltd., Taichung, Taiwan). The flies that were able to escape the tube were considered flyers and were sexed and recorded daily (except on weekends). At the end of the emergence period (approximately five days), the un-emerged pupae were recorded. The flies that were not able to escape the flight tube were classed as non-flyers. Emergence was defined as the number of emerged flies divided by the total number of initial pupae, while flight propensity was calculated as the number of flying flies divided by the number of emerged flies. Thereafter, the survival of the “flyers” was monitored daily without feeding. Ten replicates were performed for each treatment for 10 + 100 Gy and 50 + 60 Gy fractionations, while six were done for the equal fractionation of 55 + 55 Gy.

### Statistical analysis

Data were statistically analysed using R studio, version 4.4.2. Generalized linear mixed models were used with the appropriate family after checking overdispersion [11]. Overdispersed models were rebuilt by using the alternative family function. When modeling the emergence rate and flight propensity, the treatments and the pupae age on their respective full irradiation days were treated as mixed effects, while the replicates were considered random effects. The emmeans function under the emmeans package was used for the pairwise comparison between the treatments or ages. In the fractionated dose irradiation trial, age is automatically considered as a variable, based on the magnitude of the time intervals. In this study, to detect the effect of radiation treatment (single or fractionated doses), pairwise comparisons were made between treatments irradiated on the same days. To detect the effect of age, comparisons were made between different ages within each treatment. The response to radiation dose, measured as a proportion of induced sterility, was analyzed using the Kruskal–Wallis rank sum test. Prior to the analysis, the normality of the data was checked using the Shapiro–Wilk normality test. Finally, radiation dose fractionation effects on male survival time were analyzed using the Cox mixed-effects model (“coxme” function in “survival” package) fit by maximum likelihood, with the treatments as fixed effects and the replicates as random effects.

## Results

### Dosimetry

The dosimetry results indicated that the absorbed doses varied less than 5% from the target doses (Supplementary Table 1).

### Assessing adult emergence rate, survival under feeding regime, and induced sterility

**Figure 2:**
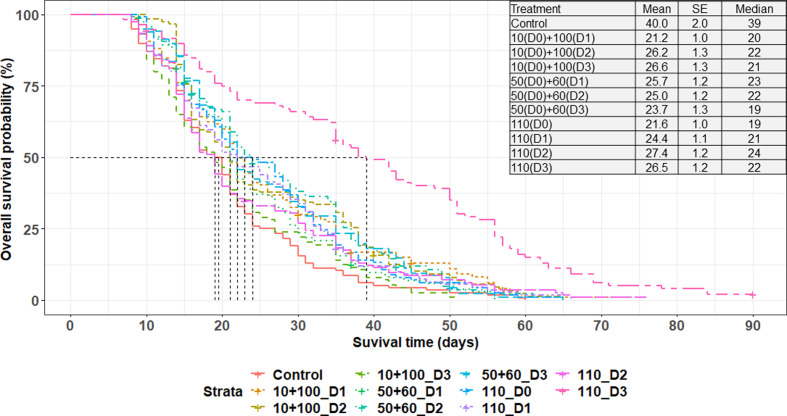
Survival of male *Glossina palpalis gambiensis* irradiated as pupae with single doses (110 Gy) as compared with those irradiated with fractionated doses of 10 + 100 Gy and 50 + 60 Gy with 1-, 2- or 3-day intervals (D1, D2, D3). Control males were not irradiated. All flies were maintained under standard feeding conditions and were monitored under feeding regime. The vertical black lines indicate the median survival time, representing the duration when the likelihood of survival decreased by 50%. The table accompanying the graphs displays the mean survival time, its standard error (SE), and the median.

**Supplementary Figure 1:**
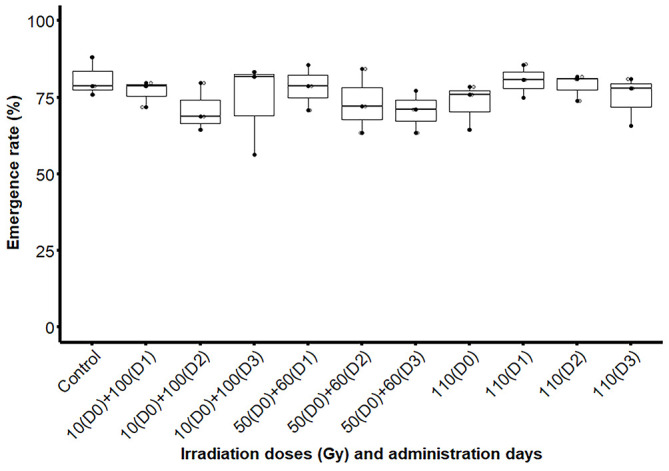
Adult emergence rate of *Glossina palpalis gambiensis* pupae irradiated with single dose (110 Gy) or fractionated doses of 10 + 100 Gy and 50 + 60 Gy with 1-, 2-, and 3-day intervals (D1, D2, D3) as compared to the non-irradiated pupae. The boxplot shows the median, upper and lower quartiles. Dots represent experimental data. There was no significant difference.

Adult emergence rates ranged from 69.2 ± 6.3% to 79.6 ± 5.0%. The generalized linear model that fitted the emergence rate data with the treatment as a fixed effect showed that there was a significant difference between the treatments (χ^2^ = 17.9130; df = 7; *p* = 0.0124). While computing a pairwise comparison using the adjusted *p*-values of the Tukey method, no significant differences were observed (Supplementary Figure 1). Neither the fractionation/single dose administration nor the age of pupae at the time of irradiation influenced the rate of adult emergence. A total of 1,427 females were available at the beginning of the 60-day survival assessment, and 1,213 flies remained at the end, resulting in an overall mortality rate of 15.0%. Survival of the males that were kept under a standard feeding regime was evaluated for 90-days and they survived on average for 25.7 ± 14.1 days. Overall, survival was significantly affected by the irradiation (χ^2^ = 101.74; df = 10; *p* < 0.0001), with the fertile untreated males surviving longer than those irradiated with a single or fractionated doses (Fig. 2). Supplementary Table 2 shows that, when considering each type of irradiation treatment (fractionated or not), males derived from pupae that received their second dose when more mature (with 2- and 3-day intervals) survived on average longer as compared with those that were irradiated with the second dose only after 1 day (10 + 100 Gy). Similarly, males irradiated with a single dose on days 26–27 post larviposition survived longer than those that received the dose on days 24–25 post larviposition. Flies irradiated with single dose survived similarly to those irradiated with fractionated doses when considering the same age on irradiation days (χ^2^ = 10.0000; df = 10; *p* = 0.4405). However, males that were exposed to 50 + 60 Gy with a 1-day interval exhibited a slightly longer survival time (25.7 ± 1.2 days) compared to those exposed to a single dose (24.4 ± 1.1 days) or a fractionated dose of 10 + 100 Gy with a 1-day interval (21.2 ± 1.0 days), as illustrated in Table 1 and Figure 2.

**Table 1:** Reproduction parameters of *Glossina palpalis gambiensis* females mated with males irradiated as pupae with fractionated (10 + 100 Gy and 50 + 60 Gy) and single doses (110 Gy), separated with 1-, 2- and 3-day intervals (D1, D2, D3). Mature female days were calculated for each treatment by adding the number of flies alive each day, starting on D18 after emergence (age of maturity) until the end of the experiment on D60.

Treatment radiation dose (Gy, days)	Parental pupae emergence (%)	Mature female days*	No. of aborted eggs and instar larvae				No. of pupae produced	Mean ± SD fecundity	Mean ± SD induced sterility (%)	Produced pupae emergence/females (%)	Age at emergence (days)
			E	I	II	III					
0	81.0 ± 6.3	4559	38	4	1	0	393	0.087 ± 0.010	0.0	94.4 (45.8)	31
10(D0) + 100(D1)	76.8 ± 4.3	4426	531	0	0	0	18	0.005 ± 0.002	95.4 ± 1.3	75.3 (44.4)	31
10(D0) + 100(D2)	71.0 ± 7.9	4985	588	1	0	0	21	0.004 ± 0.002	94.7 ± 1.9	69.1 (23.8)	32
10(D0) + 100(D3)	73.8 ± 15.2	4505	497	0	1	0	32	0.007 ± 0.005	90.5 ± 7.5	81.5 (40.6)	31
50(D0) + 60(D1)	78.4 ± 7.4	5219	582	2	0	0	18	0.003 ± 0.002	95.0 ± 4.8	67.5 (22.2)	31
50(D0) + 60(D2)	73.3 ± 10.5	5197	595	1	0	0	23	0.005 ± 0.002	94.4 ± 2.0	71.7 (34.8)	32
50(D0) + 60(D3)	70.6 ± 6.8	4931	565	0	1	0	23	0.005 ± 0.002	93.6 ± 5.0	66.4 (43.5)	31
110(D0)	73.0 ± 7.5	5385	587	3	1	0	22	0.004 ± 0.002	95.2 ± 3.5	72.0 (18.2)	31
110(D1)	80.5 ± 5.4	5052	565	5	0	0	21	0.004 ± 0.003	94.4 ± 5.0	72.9 (28.6)	32
110(D2)	78.9 ± 4.3	5496	646	2	1	0	26	0.005 ± 0.004	93.1 ± 5.4	83.3 (34.6)	30
110(D3)	74.8 ± 8.1	5011	573	2	1	0	32	0.006 ± 0.003	91.0 ± 5.4	94.0 (40.6)	31

D: day; E: egg; I: instar larvae I; II: instar larvae II; III: instar larvae III; SD: Standard deviation.

**Figure 3:**
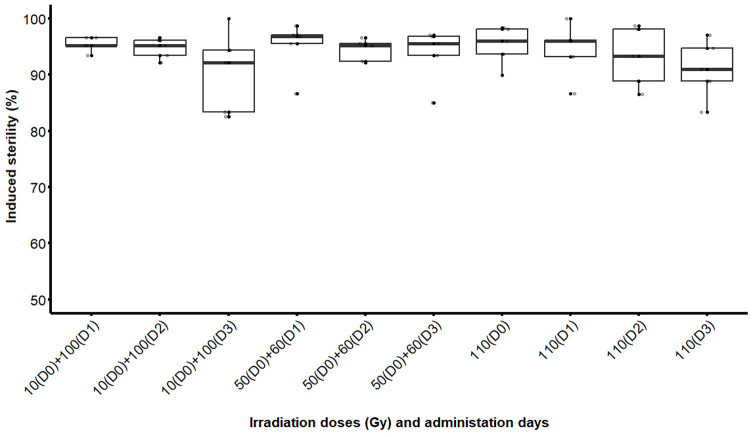
Induced sterility in female *Glossina palpalis gambiensis* mated with males that were irradiated as pupae with a single dose (110 Gy) or fractionated doses of 10 + 100 Gy and 50 + 60 Gy with 1-, 2- or 3-day intervals (D1, D2, D3). Females from the control group had mated with untreated fertile males. The boxplot shows the median and upper and lower quartiles. Dots represent experimental data.

The dose-response assessment showed that all irradiation treatments resulted in sterility levels greater than 90% as compared with the control group. Residual fertility of females mated with males irradiated with a single or fractionated doses was low, irrespective of fractionation proportions (10 + 100 Gy or 50 + 60 Gy) and intervals between exposures (0-, 1-, 2- and 3-days) (Fig. 3; Table 1). There were no significant differences in the level of induced sterility between females that had mated with males that had received a single dose, fractionated doses or between the two fractionation proportions (χ^2^ = 5.3698; df = 9; *p* = 0.8010). Across all types of irradiations, whether treatment was with a single or fractionated dose, induced sterility decreased with pupal age, as shown in Figure 3 and Table 1.

The number of eggs aborted was significantly higher in females mated with irradiated males than those mated with fertile males (Table 1), irrespective of whether the radiation doses were fractionated or given as a single dose. Dissections of the females conducted after the 60-day experimental period revealed that, regardless of whether the radiation doses were fractionated or given in a single dose, the irradiated males retained their ability to mate and transfer sperm to the females, as evidenced by the insemination rate (>90%) and the spermathecae fill (Table 2). The content of the uterus during the dissection revealed a difference between the females that had mated with fertile males and those that had mated with irradiated males. Females that had mated with fertile males had either a recently ovulated egg or a larva in the uterus, and some had recently deposited the third instar larvae (post larviposition). Conversely, females that had mated with irradiated males displayed an empty uterus and the next ovarian follicle was not yet mature, which strongly suggests that the eggs or instar larvae had recently been aborted (Table 2).

**Table 2:** Reproductive status of *Glossina palpalis gambiensis* females that mated with males exposed as pupae to fractionated (10 + 100 Gy and 50 + 60 Gy) and single (110 Gy) doses, separated by 1-, 2- and 3-day intervals (D1, D2, D3), dissected after an experimental period of 60 days.

Irradiation treatments (Gy, days)	No. of live females at day 60	Insemination rate (%)	Spermathecae fill score					Uterus content or status at day 60						
								No. of recently ovulated eggs	Viable instar larvae			Empty due to		Blockage
			0	0.25	0.50	0.75	1		I	II	III	Larviposition	Abortion	
0	95	100.0	1	1	8	64	22	19	13	8	25	26	5	0
10(D0) + 100(D1)	96	99.3	1	1	11	57	29	13	0	2	0	0	84	0
10(D0) + 100(D2)	110	96.5	4	6	6	56	37	10	1	0	2	0	95	1
10(D0) + 100(D3)	100	90.9	10	9	17	48	16	7	0	0	2	0	90	1
50(D0) + 60(D1)	116	96.7	4	3	20	63	23	16	1	0	2	0	94	0
50(D0) + 60(D2)	115	97.2	3	1	12	71	23	33	0	0	1	0	75	1
50(D + 60(D3)	110	98.9	1	2	15	69	22	14	0	1	2	0	88	4
110(D0)	121	96.7	4	2	17	59	37	21	0	0	2	0	96	0
110(D1)	113	96.2	4	2	9	62	34	11	0	1	1	0	98	0
110(D2)	126	97.5	3	1	12	56	54	12	0	0	1	0	112	0
110(D3)	111	100.0	0	0	5	63	41	11	0	0	5	0	93	0

### Evaluating flight quality using fractionated doses: adult emergence rate, flight propensity, and survival under stress regime

#### Fractionation of 10 + 100 Gy and 50 + 60 Gy

**Figure 4:**
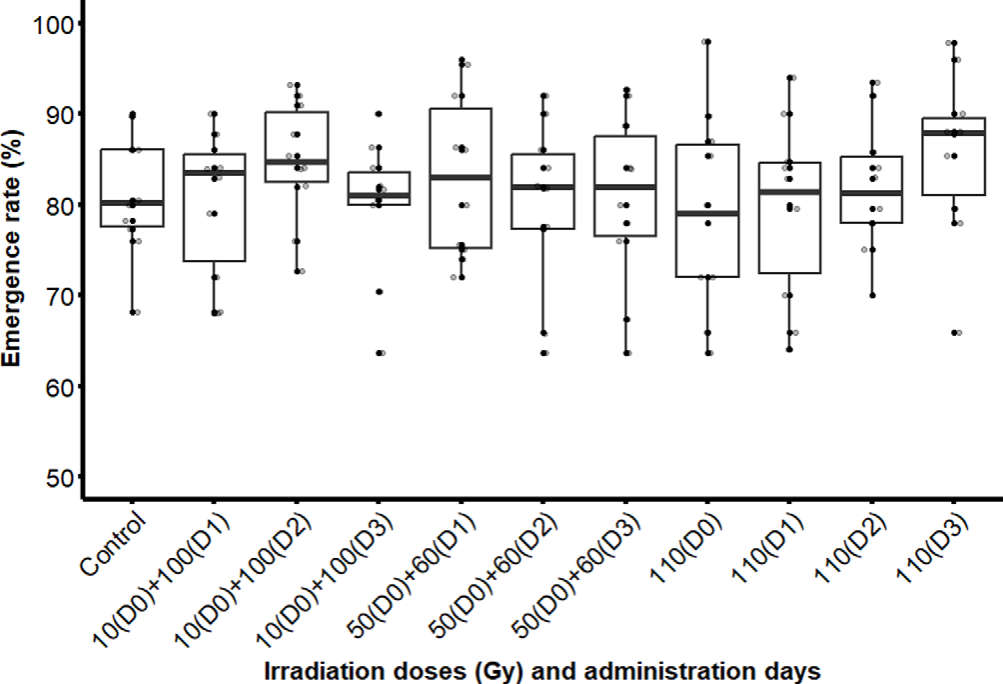
Adult emergence rate of male *Glossina palpalis gambiensis* irradiated as pupae with a single dose (110 Gy) or a fractionated dose of 10 + 100 Gy and 50 + 60 Gy with 1-, 2- or 3-day intervals. The control group consisted of non-irradiated pupae. The boxplot shows the median and upper and lower quartiles. Dots represent experimental data.

The emergence rate of pupae irradiated with a single or fractionated doses did not differ significantly, neither from the emergence rate of the pupae of the control group (Fig. 4), nor from the two types of fractionations (χ^2^ = 6.3813; df = 10; *p* = 0.7823). Thus, the type of irradiation treatment (single or fractionated dose) and the age of the pupae during the second dose (1, 2, and 3-day interval) did not have a significant impact on adult emergence rates.

**Figure 5:**
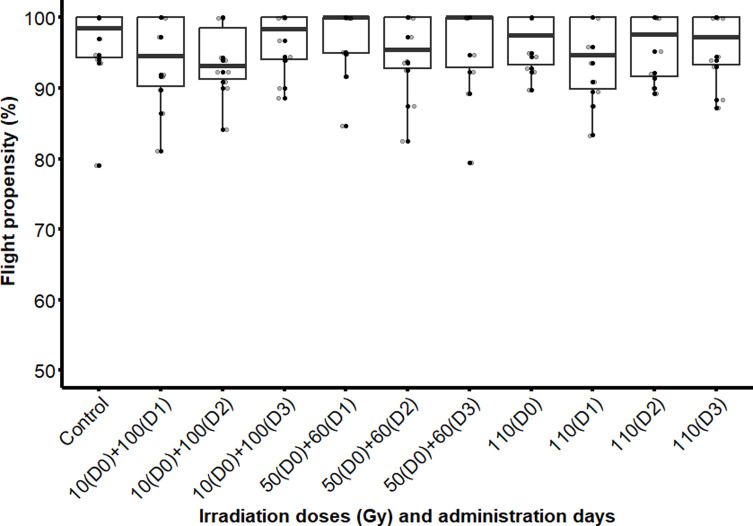
Flight propensity of male *Glosinna palpalis gambiensis* irradiated as pupae with a single dose (110 Gy) or a fractionated dose of 10 + 100 Gy and 50 + 60 Gy with 1-, 2- or 3-day intervals. The control group consisted of non-irradiated males. The boxplot shows the median and upper and lower quartiles. Dots represent experimental data.

The propensity of emerged flies to escape the flight cylinder was similar with various treatments (χ^2^ = 7.4597; df = 10; *p* = 0.6815) (Fig. 5). This means that there was no notable variation in the flight propensity of flies that emerged from pupae irradiated with either a single or fractionated doses, nor between the 10 + 100 Gy and 50 + 60 Gy fractionations, as compared with the control group. These findings were consistent across all irradiation time intervals. However, it should be noted that a slightly higher propensity to fly was observed in flies irradiated with fractionated doses of 50(D0) + 60(D1) Gy with a 1-day interval as compared with those irradiated with a single or fractionated doses of 10 + 100 Gy with the same time interval and a single dose on day 0.

**Figure 6:**
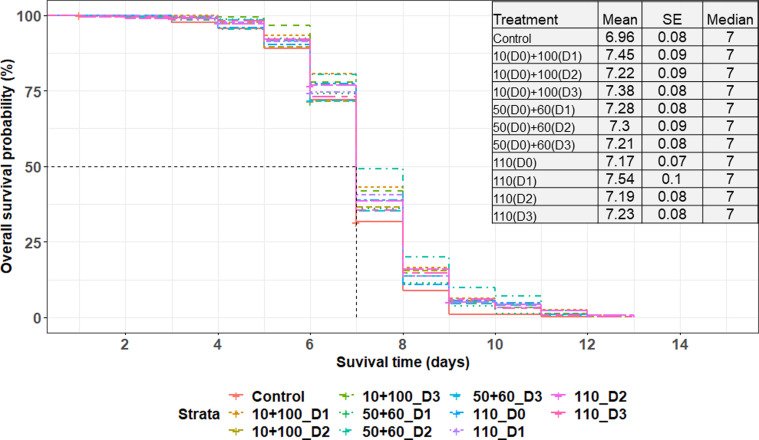
Survival curves of male *Glossina palpalis gambiensis* irradiated as pupae with a single (110 Gy) or fractionated doses of 10 + 100 Gy and 50 + 60 Gy, separated by 1-, 2- and 3-day intervals (D1, D2, D3). The control group consisted of non-irradiated males. All flies were maintained under standard rearing conditions and were monitored under feeding stress. The black vertical line indicates the median survival time, representing the duration when the likelihood of survival decreased by 50%. The table accompanying the graphs displays the mean survival time, its standard error (SE), and the median.

As for survival under feeding stress, there was an overall significant difference between the treatments (χ^2^ = 36.9590; df = 10; *p* = 5.753e-05 (Fig. 6). However, pairwise comparison of the survival of flies that emerged from pupae irradiated with both fractionation types and the single dose on the same day did not reveal any statistically significant differences (Supplementary Table 3). Nevertheless, regarding pupae age in each treatment, flies that emerged from pupae irradiated with a single dose on day 25–26 post larviposition showed significantly higher survival rates than those that emerged from pupae irradiated with a single dose on day 24–25 post larviposition and day 26–27 post larviposition (Supplementary Table 4). In addition, males irradiated with a single dose or a fractionated dose of 10 + 100 Gy with a 1-day interval survived longer than the fertile males (Supplementary Table 5).

#### Equal fractionation of 55 + 55 Gy

**Figure 7:**
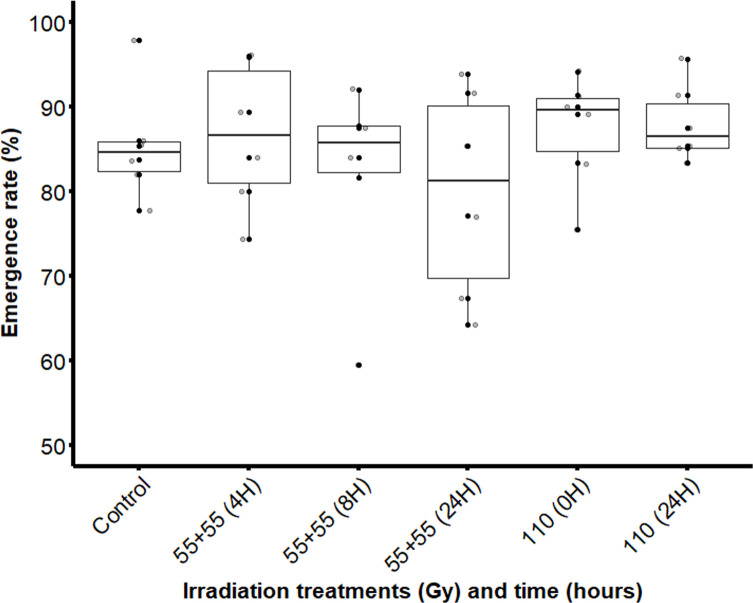
Adult emergence rate of male *Glossina palpalis gambiensis* irradiated as pupae with a single dose (110 Gy) or equal fractionated doses of 55 + 55 Gy with 4-, 8- or 24-hour intervals. The control group consisted of non-irradiated pupae. The boxplot shows the median and upper and lower quartiles. Dots represent experimental data.

Splitting the optimal radiation dose into two equal doses of 55 Gy separated by 4-, 8- and 24-hour intervals did not significantly affect the adult emergence rate and there were no significant differences between the irradiated and the non-irradiated groups (χ^2^ = 3.6979; df = 5; *p* = 0.5937) (Fig. 7).

**Figure 8:**
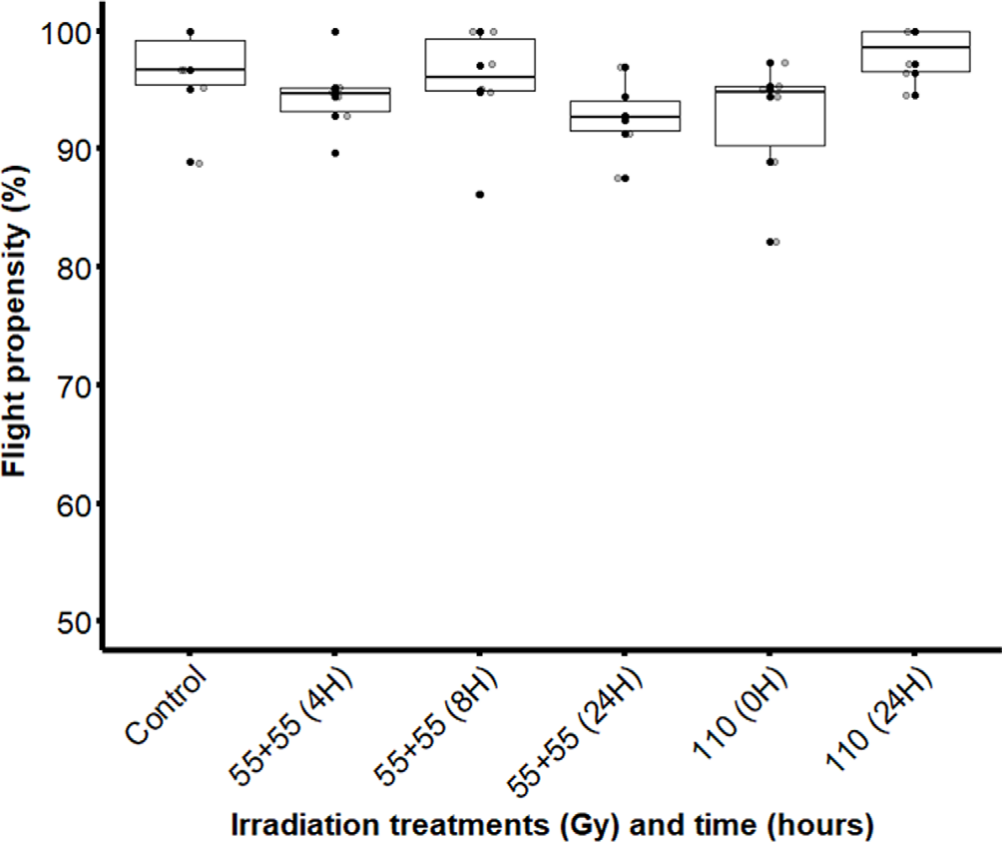
Flight propensity of male *Glossina palpalis gambiensis* irradiated as pupae with a single dose (110 Gy) or equal fractionated doses of 55 + 55 Gy with 4-, 8- or 24-hour intervals. The control group consisted of non-irradiated males. The boxplot shows the median and upper and lower quartiles. Dots represent experimental data.

Similar to the adult emergence rate, splitting the optimal radiation dose into two equal doses did not significantly impact the flight propensity (χ^2^ = 8.8826; df = 5; *p* = 0.1138) (Fig. 8).

**Figure 9:**
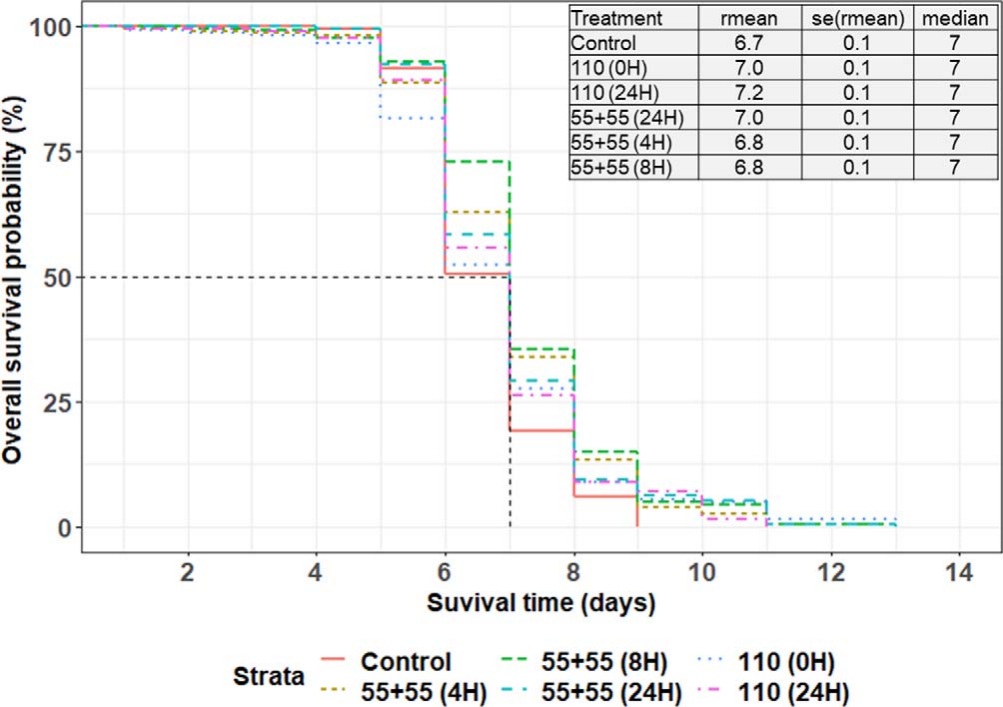
Survival curves under feeding stress of male *Glossina palpalis gambiensis* irradiated as pupae with a single (110 Gy) or equal fractionated doses of 55 + 55 Gy, separated by 4-, 8- and 24-day intervals. The control group consisted of non-irradiated males. All flies were maintained under standard rearing conditions and were monitored under feeding stress. The black line indicates the median survival time, representing the duration when the likelihood of survival decreased by 50%. The table accompanying the graphs displays the mean survival time, its standard error (SE), and the median.

Under feeding stress, the median survival time, representing the duration when the likelihood of survival decreased by half across all treatments, was observed to be 7 days. The Cox mixed-effects model fit by maximum likelihood showed that the survival time varied significantly across the treatment (χ^2^ = 23.656; df = 5; *p* = 0.0003). Pairwise comparison using the Tukey method for adjusting *p*-values showed that males from pupae irradiated with a single dose of 110 Gy at 0 h and 24 h after sorting, as well as those irradiated with equal fractionated doses of 55 Gy separated by 24 h, survived significantly longer than the non-irradiated group (Fig. 9, Supplementary Table 6). Nevertheless, as illustrated in the first fractionations, there was no notable difference in the survival rates of male individuals emerging from pupae irradiated with either a single dose or equal fractionated doses, regardless of the time interval between the two equal doses (Supplementary Table 6).

## Discussion

Sterilization of insects using ionizing radiation is a crucial step in the successful implementation of the SIT as part of AW-IPM programs [4]. Research and development (R&D) plays a crucial role within the framework of these programs, as they are essential in improving the cost-effectiveness of all aspects of the SIT application, including radiation biology [60]. Through R&D, protocols can be refined and optimized, resulting in enhanced program efficiency and effectiveness [13]. These efforts aim to advance the SIT application and related technologies, leading to their wider adoption and use. Insect radiation protocols are continuously improved under the SIT operational program framework. Aspects that were recently or are currently being investigated are the feasibility of using the SIT as a new pest management method for several new species [8, 18, 23], the use of X-rays as an alternative to gamma rays [23, 65, 69], the effect of radiation dose rate on the biological response [38, 70], the effect of radiation on insects symbionts of interest [22, 40], the radiation sensitivity of the different live stages of insects [3, 45], and the effect of dose fractionation [67] and of different atmospheric and environmental conditions on radiation sensitivity and somatic damage [9]. The objective of these studies is to optimize sterilization protocols to provide the highest level of sterility with the lowest level of somatic damage [31, 46]. The positive effect of irradiation fractionation on sterility has been demonstrated in several insects species [62, 67].

In the current study, *G. p. gambiensis* pupae were irradiated with a dose of 110 Gy that was recommended as optimal for this species some 50 years ago [34, 54], either as a single dose or as fractionated doses, and the effects were assessed on induced sterility and flight quality. Two types of fractionations were selected based on previous studies [15, 67]: for the first, the doses were almost an equal split of the optimal dose (50 + 60 Gy), while for the second, the first dose administered was low (10 Gy) followed by a high dose (100 Gy). The different fractionations were separated by 1-, 2-, and 3-days. The results obtained revealed that the rate of induced sterility by a single or fractionated doses did not significantly differ, regardless of the time intervals. Similar results were obtained with *G. tachinoides* when younger pupae (15 days post larviposition) were irradiated with fractionated doses under a nitrogen atmosphere [59]. In contrast, this result differs from that of Yamada *et al.* [67] which showed that fractionated doses led to complete sterility in *Aedes aegypti* that mated with irradiated males [67]. Dose fractionation assessments with the Indian meal moth *P. interpunctella* showed that the percentage of sterile pairs was dependent both on the dose and on the pattern of fractionation [14]. LaChance and Graham reported that exposing *Musca domestica* L. (housefly) (Diptera), *Oncopeltus fasciatus* (Dallas) (milkweed bug) (Hemiptera), *Anagasta kuehniella* (Zeller) (meal moth) (Lepidoptera), and *Heliothis virescens* (Fab.) (Tobacco budworm) (Lepidoptera) to a single dose of radiation or fractionated into two equal exposures separated by an 8-hour interval did not decrease the frequency of induced lethal mutations [37]. These observations suggest that the time interval might be a crucial parameter when doses are fractionated. The inconsistency of the data from the different studies may be explained by the species and developmental stage-specific impact of radiation on insects. The sensitivity of insects to the induction of dominant lethal mutations depends on their developmental stage [5, 28, 33], necessitating the identification of a suitable radiation dose that can effectively achieve the desired level of sterility while preserving the overall quality of the released insects [49]. Previous dose-response studies with tsetse species showed a different sensitivity when pupae or adults were irradiated [6, 7, 42, 44, 54]. In the current study, it was clear that irradiating *G. p. gambiensis* pupae using any of the dose fractionation combinations resulted in a decrease of induced sterility with increasing age. The reason for these age- or development stage-related differences may be attributed to changes in age-dependent gene expressions. Several genes involved in resistance to toxic chemicals were upregulated in aging *Drosophila*, reflecting an overall increase in the defense response system with age [12]. Moreover, our results showed that there was no significant difference in reproductive parameters such as the rate of egg abortion, insemination rate, and the presence of sperm in the spermathecae of females mated with males from pupae exposed to a single or fractionated doses, irrespective of the time intervals between the fractionations. Our results showed similarities with the results obtained from an experiment carried out on *G. tachinoides*, where fractionated doses were administered during the mid-pupal stage [59]. When assessing the impact of dose fractionation on adult emergence rate, flight propensity, and survival time, there were no significant differences between the fractionations and the single dose, nor between the two types of fractionated doses given in the same time interval. It was hypothesized that the phenomenon of radiation hormesis would lead to an expected improvement in lifespan as exposing an organism or cell to low doses of an agent can produce positive biological responses, whereas a higher dose of the same agent can decrease the beneficial effects [16]. However, contradictory results available in the literature provide uncertainty that hormesis is a “real phenomenon”. Ionizing radiation administered to the nematode *Caenorhabditis elegans* did not promote subsequent resistance or increased longevity [20], while the results are different from those obtained on insects species such *Ae. aegypti* [67], *P. interpunctella* [14]*,* and adult *Spodoptera litura* [56]*.* The observed increase in longevity of *G. tachinoides* when exposed to gamma radiation may be attributed to the additional nitrogen atmosphere present during irradiation [59], since radiation under hypoxic or anoxic conditions could improve the insects’ biological parameters [55]*.* For flight propensity, our results were similar to those found for *Aedes aegypti* [67]*.* From these observations, the contrasting results in the hormesis investigations are believed to be the result of experimental design considerations, particularly with respect to the number of doses, the range of doses, and the selection of the endpoint [16]. Overall, while our results did not indicate any significant difference in the parameters assessed, male from pupae exposed to a fractionated dose of 50 + 60 Gy with a 1-day interval demonstrated a marginally higher level of induced sterility, flight propensity, and survival time (under feeding), when compared to those subjected to either a single dose or a fractionated dose of 10 + 100 Gy. These observations imply that the values of the fractionation and the time intervals between exposures may play a significant role in determining the response of irradiated insects. Among the available studies on fractionated-dose irradiation, although the methods used vary, the majority have employed either one low and one high dose, or two equal doses. The biological stage and the time interval between the administration of fractionated doses could be a crucial factor in determining the response of the irradiated pupae. Depending on the stage of spermatogenesis, giving a single or fractionated dose may have the same effect on the final sterility. Alexander and Bergendahl [1] demonstrated that exposure of adult *Drosophila virilis* to single or consecutive low doses had no significant effect on sterility when the spermatozoa were mature. However, when the dose was fractionated at the spermatid stage, an increase in genetic damage was observed, leading to an increase in sterility [1]. Regarding the time intervals between the administration of doses and biological stage, selecting the appropriate development stage and intervals between exposures is crucial. However, the optimal timing for this species remains unknown, especially due to the limited research on fractionated radiation doses in tsetse flies. The interval between two administrations of radiation at 1, 2, and 3 days may be too long, which can affect the immune system’s ability to remain active depending on the radiation type. When the germ cells are mature and their metabolism is inactive during irradiation, recovery mechanisms cannot function. This also holds true for somatic cell damage, which increases with dose and decreases when irradiation occurs at a later stage of insect development, as the number of dividing cells decreases [31]. This is especially true as irradiation affects not only the chromatin material but also partially blocks metabolic pathways [48]. However, the same results were not obtained with the fractionated doses of 10 + 100 Gy or with the single dose on day 1 compared to that of 50 + 60 Gy, indicating the importance of the required dose to stimulate an appropriate response in the insect organism. The 10 Gy dose administered on day zero might be too low, as low doses have been known to have a double effect, including a low probability of damage and adaptive protection against DNA damage through prevention and repair, as well as immune stimulation [29], since the fractionation is supposed to stimulate somatic cell recovery between doses [25]. As hormetic radiation doses vary considerably between species, we conducted an additional experiment to explore the effects of equal fractional doses of 55 + 55 Gy separated by intervals of 4, 8, and 24 h. Once again, no significant effects were observed. The comprehensive outcomes of our study emphasize the potential need for further investigation, as exemplified by studies conducted by Vimal *et al.* [56] or exploration of the synergy between dose fractionation and a low-oxygen atmosphere. In addition, the biological development stage should be considered. Since previous results indicate that fractionating doses on earlier pupae stages in the presence of nitrogen greatly improved *G. tachinoides* longevity [59], biological stage, exposures interval time, and radiation atmosphere should be considered in future studies. An important notion to consider when selecting the developmental stage to evaluate is the current pupal sex sorting time constraints [2]. In the absence of a genetic sexing strain for *G. p. gambiensis*, the technology of the NIRPSS can only separate pupae according to their sex at an advanced stage of development. Selecting male pupae at the beginning or in the middle of their pupal development is currently not possible. Consequently, the practice of dose fractionation might be challenging to implement in SIT operational programs. For instance, during routine irradiation and shipment of pupae, which occurred twice a week for the Senegal eradication project – specifically on Tuesdays for pupae sorted from Saturday to Tuesday, and on Fridays for pupae sorted from Wednesday to Friday – it would have been complex to implement dose fractionation under these schedules. Furthermore, dose fractionation would result in a doubling of the workload for the radiation operator, as well as repeated handling of the pupae that may cause stress [19, 66]. Thus, this increased workload and likely stress on the pupae must be carefully weighed against the potential benefits in terms of improved sterile male quality parameters.

## Conclusion

The findings of our study suggest that the value of hormesis induced in fractionally sterilized males is influenced by the pupal developmental stage and fractionated doses, which is consistent with previous studies. The biological benefits generated by dose fractionation in this study are relatively modest when compared to the associated workloads and transportation constraints. As a result, it is challenging to recommend its immediate implementation without further considerations and optimizations. Alternative dose fractionation schemes could be investigated in the future to determine if there are more efficient ways to achieve better results, while reducing workload. Although current benefits may be limited, exploration of alternative methods, such as irradiation in a low-oxygen environment, may pave the way for more effective and practical irradiation protocols.

## Data Availability

Materials described in the paper, including all relevant raw data, are available in this link: https://doi.org/10.7910/DVN/TJ7NOO.

## Supplementary materials

The Supplementary materials of this article are available at https://www.parasite-journal.org/10.1051/parasite/2024001/olm

*Supplementary Table 1*: Overview of *Glossina palpalis gambiensis* radiation dosimetry data using the GAFchromic dosimetry system. Data were summarized based on radiation dose and days. The first fraction of the dose was given on Day 0 or hour 0 followed by the second part on day 1, 2 and 3 or after 4 hours, 8 hour and 24 hours. The single doses were given from day 0 to day 3 or at hour 0 and 24 hours. The data were averaged. *Supplementary Table 2*: Pairwise comparison of the survival time of *Glossina palpalis gambiensis* males emerged from non-irradiated pupae and those irradiated with single dose (110 Gy) or fractionated doses of 10 + 100 Gy and 50 + 60 Gy with 1-, 2-, 3-day intervals (D1, D2, D3). Survival was monitored under conditions of feeding regime. *Supplementary Table 3*: Pairwise comparison of survival time under feeding stress of male *Glossina palpalis gambiensis* from pupae irradiated with single dose (110 Gy) and fractionated doses of 10 + 100 Gy and 50 + 60 Gy separated by 1-, 2- and 3-day intervals (D1, D2, D3). Survival was monitored under conditions of feeding stress. The comparison was based on the same age to detect the possible effect of the irradiation treatments (single or fractionated doses). *Supplementary Table 4*: Pairwise comparison of survival time under feeding stress of male *Glossina palpalis gambiensis* from pupae irradiated with single dose (110 Gy) and fractionated doses of 10 + 100 Gy and 50 + 60 Gy separated by 1-, 2- and 3-day intervals (D1, D2, D3). Survival was monitored under conditions of feeding stress. The comparison was based on the same irradiation treatment to detect the effect of the age of the pupae at the day of irradiation. *Supplementary Table 5*: Pairwise comparison of the survival time of *Glossina palpalis gambiensis* males emerged from non-irradiated pupae and those irradiated with single dose (110 Gy) or fractionated doses of 10 + 100 Gy and 50 + 60 Gy with 1-, 2-, 3-day intervals (D1, D2, D3). Survival was monitored under conditions of feeding stress. *Supplementary Table 6*: Pairwise comparison of the survival time of male *Glossina palpalis gambiensis* emerged from non-irradiated pupae and those irradiated with a single dose (110 Gy) and equal fractionated doses of 55 + 55 Gy separated by 4-, 8- and 24-hour intervals. Survival was monitored under conditions of feeding stress.
